# Hyperemic stress myocardial perfusion cardiovascular magnetic resonance in mice at 3 Tesla: initial experience and validation against microspheres

**DOI:** 10.1186/1532-429X-15-62

**Published:** 2013-07-21

**Authors:** Roy Jogiya, Markus Makowski, Alkystsis Phinikaridou, Ashish S Patel, Christian Jansen, Niloufar Zarinabad, Amedeo Chiribiri, Rene Botnar, Eike Nagel, Sebastian Kozerke, Sven Plein

**Affiliations:** 1 King’s College London BHF Centre of Excellence, NIHR Biomedical Research Centre and Welcome Trust and EPSRC Medical Engineering Centre at Guy’s and St. Thomas’ NHS Foundation Trust, Division of Imaging Sciences, The Rayne Institute, London, UK; 2 Academic Department of Surgery, Cardiovascular Division, BHF Centre of Excellence, Kings College, London, UK; 3 Institute for Biomedical Engineering, University and ETH Zurich, Zurich, Switzerland; 4 Multidisciplinary Cardiovascular Research Centre & Leeds Institute of Genetics, Health and Therapeutics, University of Leeds, Leeds, LS2 9JT, UK

**Keywords:** Cardiovascular magnetic resonance imaging, Myocardial perfusion, Murine

## Abstract

**Background:**

Dynamic first pass contrast-enhanced myocardial perfusion is the standard CMR method for the estimation of myocardial blood flow (MBF) and MBF reserve in man, but it is challenging in rodents because of the high temporal and spatial resolution requirements. Hyperemic first pass myocardial perfusion CMR during vasodilator stress in mice has not been reported.

**Methods:**

Five C57BL/6 J mice were scanned on a clinical 3.0 Tesla Achieva system (Philips Healthcare, Netherlands). Vasodilator stress was induced via a tail vein catheter with an injection of dipyridamole. Dynamic contrast-enhanced perfusion imaging (Gadobutrol 0.1 mmol/kg) was based on a saturation recovery spoiled gradient echo method with 10-fold k-space and time domain undersampling (*k-t* PCA). One week later the mice underwent repeat anaesthesia and LV injections of fluorescent microspheres at rest and at stress. Microspheres were analysed using confocal microscopy and fluorescence-activated cell sorting.

**Results:**

Mean MBF at rest measured by Fermi-function constrained deconvolution was 4.1 ± 0.5 ml/g/min and increased to 9.6 ± 2.5 ml/g/min during dipyridamole stress (P = 0.005). The myocardial perfusion reserve was 2.4 ± 0.54. The mean count ratio of stress to rest microspheres was 2.4 ± 0.51 using confocal microscopy and 2.6 ± 0.46 using fluorescence. There was good agreement between cardiovascular magnetic resonance CMR and microspheres with no significant difference (P = 0.84).

**Conclusion:**

First-pass myocardial stress perfusion CMR in a mouse model is feasible at 3 Tesla. Rest and stress MBF values were consistent with existing literature and perfusion reserve correlated closely to microsphere analysis. Data were acquired on a 3 Tesla scanner using an approach similar to clinical acquisition protocols, potentially facilitating translation of imaging findings between rodent and human studies.

## Background

Myocardial blood flow (MBF) is one of the most relevant parameters in cardiovascular disease. While coro nary autoregulation maintains resting MBF constant over a wide range of physiological and pathological states, MBF reserve is impaired in several disease processes including atherosclerosis [1], systemic hypertension [2] and diabetes mellitus [3]. Rodent models play a key role in developing our understanding of cardiovascular disease and the development of novel therapies [4,5]. Accurate non-invasive assessment of MBF and MBF reserve in rodent models of cardiovascular disease is therefore highly desirable. Currently used methods include nuclear perfusion imaging [6], echocardiography [7], and spin labelling cardiovascular magnetic resonance (CMR) [8]. However, nuclear and echocardiographic perfusion methods are limited by relatively low spatial resolution and spin labelling CMR methods require long acquisition protocols. In human studies, dynamic contrast-enhanced myocardial perfusion imaging during vasodilator stress is the method of choice for the detection of ischemia with CMR. Clinical studies have demonstrated high diagnostic accuracy of the method for the detection of coronary artery disease [9,10]. While in clinical settings, data are usually interpreted by visual analysis, estimations of MBF and MBF reserve can also be derived from the acquired data [11,12]. Such quantitative analysis methods increase the objectivity of the analysis and allow monitoring of disease progression and assessment of treatment efficacy, which is an important consideration in developing translatable rodent models of cardiovascular disease. In rodents, dynamic contrast-enhanced myocardial perfusion CMR is challenging because of the high temporal and spatial resolution requirements. Making use of new data acquisition acceleration schemes, quantitative murine first pass myocardial perfusion at rest has recently been described [13-15]. Hyperemic myocardial first pass stress perfusion CMR in mice has however not been reported or validated against microspheres.

The aim of this study was to test the feasibility of myocardial perfusion CMR in C57BL/6 J mice during dipyridamole-induced hyperemia and validate the method against the reference test of injectable microspheres.

## Methods

### Animal model

Five 6-month-old homozygous C57BL/6 J male mice (weight 25-30 g) were acquired from Charles Rivers Laboratories (Edinburgh, UK) and housed within the Animal Unit of the Rayne Institute at King’s College London. The housing and care of the animals and all the procedures used in these studies were performed in accordance with the guidelines and regulations of the United Kingdom Home Office under the Animals (Scientific Procedures) Act, 1986.

### Preparation and sedation

Mice were anesthetized and maintained under inhalational anesthesia via a nose cone (1.5% isoflurane/medical oxygen). Rectal temperature was monitored continuously and a warm air flow (using an MR-compatible heater system; SA Instruments, Stony Brook, NY) was adapted to maintain temperature at 37°C. All experiments were conducted at a similar time of the day and in the same climate conditions. Two ECG leads (SA Instruments, Stony Brook, NY) were placed subcutaneously on the left and right side of the thorax. For electrocardiogram (ECG) synchronization a dedicated small-animal ECG device, 1025-MR (SA Instruments, Stony Brook, NY) and for signal reception, a microscopy receive coils (23 mm diameter, single circular loop; Philips Healthcare, Hamburg, Germany) was used.

### CMR protocol

Imaging was performed on a clinical 3.0-T system (Achieva; Philips Healthcare, Best, The Netherlands) equipped with a clinical gradient system (30 mT/m; 200 mT/m/ms). Mice were imaged under isofluorane anesthesia and in prone position. The in-built ECG triggering unit of the CMR scanner was modified to permit synchronization of heart rates of up to 600 beats/min.

All data were acquired during free breathing of the animals. For localization of the heart, low-resolution gradient echo scout scans were acquired in the coronal and transverse orientations followed by pseudo two- and four-chamber gradient echo cine scans acquired with a prospective ECG-triggered spoiled gradient echo sequence (field of view 35 × 35 mm^2^, matrix 160, slice thickness 1 mm, 5 fold k-t *SENSE* acceleration with 11 interleaved training profiles, pulse repetition time/echo time 12/6.3 msec, flip angle 20°). From these images, a true mid-ventricular short-axis view of the left ventricle was planned for subsequent perfusion imaging.

### MR perfusion pulse sequence

The perfusion pulse sequence parameters were similar to a previously published method [15]: two-dimensional saturation recovery spoiled gradient echo (Turbo Filed Echo/TFE), repetition time/echo time 6.7 msec/1.0 msec, flip angle 20°, 10-fold *k-t* undersampling with three densely sampled training profiles interleaved with the undersampled data, 62.5% partial Fourier and partial echo acquisition, one slice acquired during each RR interval, field of view 25 × 25 mm^2^, slice thickness 1.5 mm, matrix 128 × 128, spatial resolution 0.2 × 0.2 mm^2^ (reconstructed to 0.13 × 0.13 mm^2^), preparation pulse delay 100 msec (to the centre of k-space), acquisition window 43 msec. The preparation pulse delay was reduced if required to enable acquisition at higher heart rates, particularly when during stress. Data were acquired for 200 heart beats. To improve temporal fidelity of the data at the high undersampling factors, *k-t* principal component analysis (*k-t* PCA) was used for image reconstruction [16].

### Contrast delivery

A customised catheter was placed in the tail vein of the mouse and 0.1 mmol/kg bodyweight Gadobutrol (Gd-DTPA) (Gadovist, Bayer, Germany) was injected after the perfusion scan was started (volume of contrast injection 2.5 to 3.0 μL based on the weight of the mouse). To ensure a reproducible bolus injection, the contrast agent, as well as 25 μL of saline, was preloaded into small-bore tetrafluoroethylene tubing and injected manually.

### Stress agent

For stress perfusion imaging, dipyridamole (Persantine, Boehringer Ingelheim, UK) 0.56 mg/kg was injected via a tail vein catheter 1 minute prior to the perfusion acquisition and into the same tail vein catheter as used for contrast delivery. Contrast injection was performed in the same standardized way as for rest scans. Stress and rest scans were performed in random order to minimize any potential bias from residual contrast agent and were separated by 15 minutes. Following imaging of stress perfusion, a slow hand injection of intravenous aminophylline (Hospira, UK ltd, 5 mg/kg diluted in a 25 μL saline flush) was given in order to minimize any effects of dipyridamole on subsequent estimation of resting blood flow. The total volume of injection during CMR perfusion imaging was 105-106 μL.

### CMR analysis

Image quality was scored from 1 to 4 (1 = uninterpretable, 2 = satisfactory, 3 = good, 4 = excellent). Quantitative analysis of MBF was performed using custom prototype image analysis software (Philips Healthcare, Best, The Netherlands). Endocardial and epicardial contours were drawn on images with best blood to myocardium contrast and copied to all other dynamic images. Any subendocardial dark rim artifacts were excluded from the contours. The myocardium was divided into four eqiangular sectors, starting from a reference point placed at the anterior septal insertion of the right ventricle. To obtain the arterial input function, a region of interest was drawn inside the left ventricular (LV) blood pool. Signal intensity (SI)/time curves were generated for the LV blood pool, for the four myocardial sectors and an average for the myocardium as a whole. The maximal upslope of the profiles was generated using best-point fitting. SI profiles were then generated for each sector and the region of interest in the LV blood pool. Enhancement ratio of signal increase and normalized SI upslope ratios between the blood pool and myocardium were calculated as (enhancement ratio = (SI max – SI baseline)/SI baseline) and (normalized SI upslope = upslope myocardium/LV).

In addition, absolute MBF was computed from the LV blood pool and myocardial tissue SI vs. time curves, using in-house software, with previously described methods based on Fermi constrained deconvolution [17].

### Microspheres and confocal microscopy

One week after MR scanning, all 5 mice mice underwent repeat anesthesia and injection of fluorescent microspheres at dipyridamole hyperemic stress and at rest in the same order and using the same stress regime as for the CMR scans. 25 μl (1.0 × 10^6^ beads/ml) of yellow-fluorescent microspheres (10 μm diameter, Invitrogen, NY) (excitation/emission 515/534 nm) were injected at rest. One minute following dipyridamole stress, 25 μl (1.0 × 10^6^ beads/ml) scarlet (excitation/emission 645/680 nm) microspheres were injected, followed by a slow hand injection of intravenous aminophylline (5 mg/kg diluted in a 25 μL saline). The total volume of injection for the microsphere experiment was 77.8-78.4 μL.

Animals were sacrificed and the myocardium stored in phosphate buffer saline. To match the CMR protocol, a 1.5 mm mid ventricular slice was later sectioned using a vibratome to obtain 150 μM sections. Ten individual slices were analysed and the fluorochromes identified and enumerated manually using confocal microscopy (Leica TCS SP5; Deerfield, IL) to establish the absolute number of microspheres injected at rest and stress. This was used to establish a myocardial perfusion reserve (MPR = total number of spheres detected at stress/total number of spheres at rest) for each mouse.

### Flow cytometry

The remaining myocardium was processed using standardised methods [18] and analysed using flow cytometry to establish the ratio of microspheres at stress and rest. Briefly, the hearts were digested by incubation in a cocktail of collagenase IV, DNAse and hyaluronidase at 37°C for 1 h, followed by trituration and filtration through a 30 μM nylon mesh. The relative number of microspheres in each cell suspension was measured on FACS Canto II flow cytometer (BD biosciences, UK) FACS Canto and analysed using FlowJo software (TreeStar Inc). The flow cytometer was calibrated to detect the spheres in accordance with their excitation/emission wavelengths using the allophycocyanin (APC) and fluorescein isothiocyanate channels (FITC). The perfusion ratio was calculated by measuring the relative number of microspheres acquired in each channel.

### Statistics

Statistical analysis was performed using SPSS 19.0 (SPSS, Inc, Chicago, IL, USA). All data were expressed as mean ± standard deviation. For comparing hemodynamics and continuous variables a *t*-test was used. For linear regression a Pearson’s correlation coefficient was used and for comparison of multiple variables a one-way analysis of variance (ANOVA) was used. Bonferroni post-hoc analyses were used to compare the calculation of the perfusion reserve using different techniques. A Bland-Altman analysis was performed to determine the mean bias and limits of agreement between CMR and microsphere estimates of MPR. Statistical significance was considered for P < 0.05 and high significance was considered for P < 0.01.

## Results

All mice tolerated the scanning without complication and rest/stress first-pass myocardial perfusion imaging was successfully performed. The mean overall imaging time was 34.6 minutes (± 7.4 minutes).

### Stress perfusion imaging

Dipyridamole was successfully injected in all 5 mice via the tail vein. The mean heart rate increased following the injection, but not to a statistically significant degree (rest perfusion: 480 ± 27 beats/min, stress perfusion 503 ± 42 beats/min, p = 0.08).

### Visual interpretation of perfusion images

**Figure 1:**
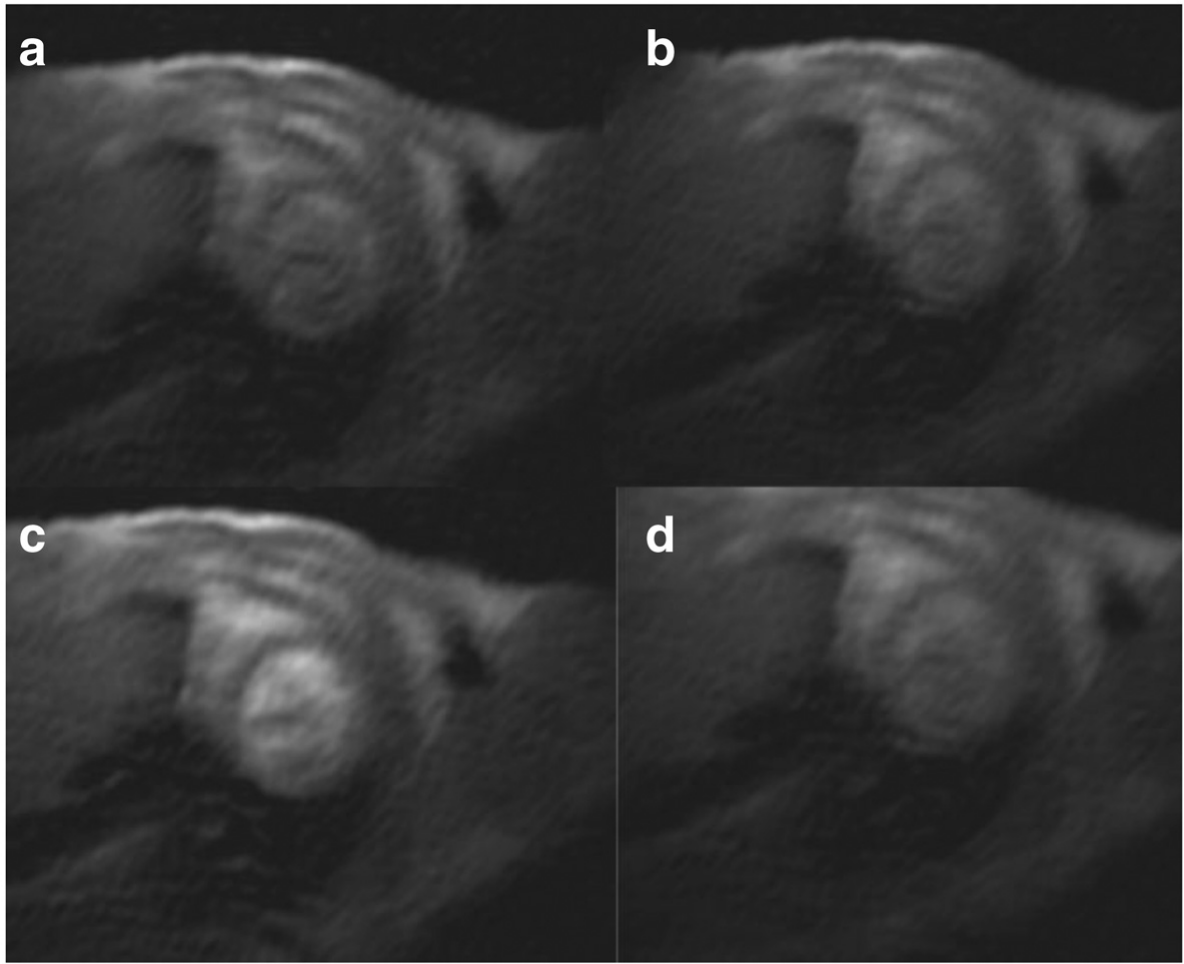
**Series of dynamic images after contrast bolus injection (0.1 mmol/kg body weight Gd-DTPA) in a short axis section of a mouse.** Spatial resolution was 0.2 x 0.2 mm^2^ (reconstructed to 0.13 × 0.13 mm^2^). Images demonstrate the baseline scan **(a)**, as well as the passage of the contrast agent in the RV **(b)**, LV **(c)**, and in the myocardium **(d)**.

Overall image quality was 2.6 ± 0.5 and quality was sufficient to outline myocardial contours for quantitative analysis in all studies (Figure 1). Breathing did not pose a significant problem as respiratory motion correction is incorporated into the *k-t* PCA algorithm. Typical transient subendocardial dark rim artifacts were noted in 2 studies, affecting in particular the inferolateral segments. These banding artifacts persisted for three to seven heartbeats during peak signal in the LV and affected 30–60% of the myocardial thickness.

### Perfusion measurements

**Figure 2:**
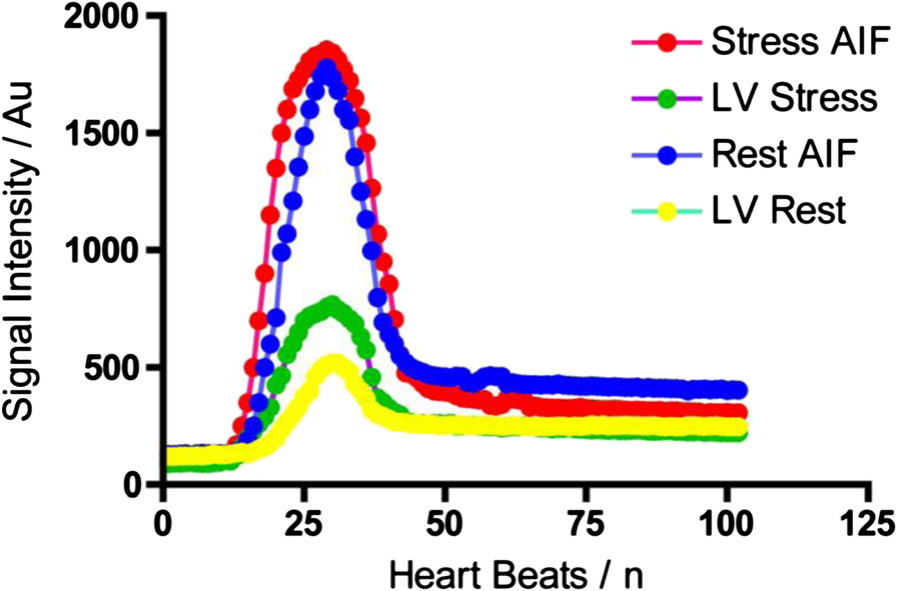
**Mean SI/phase profile from five mice at rest and stress.** The different color codes represents the passage of the contrast agent (CA) within the LV and myocardial cavity.

In three animals, stress imaging was performed first, and in two animals rest was performed first. Signal intensity/time profiles derived during stress and rest showed similar features to human profiles (Figures 2). At rest, mean LV signal increased from 129 ± 24 (arbitrary units) before contrast delivery to a peak of 1777 ± 253, resulting in an enhancement ratio of 13.3. Mean myocardial signal increased from 120 ± 6 before contrast to 518 ± 79, with an enhancement ratio of 3.3. The mean resting MBF by Fermi-constrained deconvolution across the four myocardial segments was 4.1 ± 0.5 mL/g/min. There were no significant differences between the segments.

**Figure 3:**
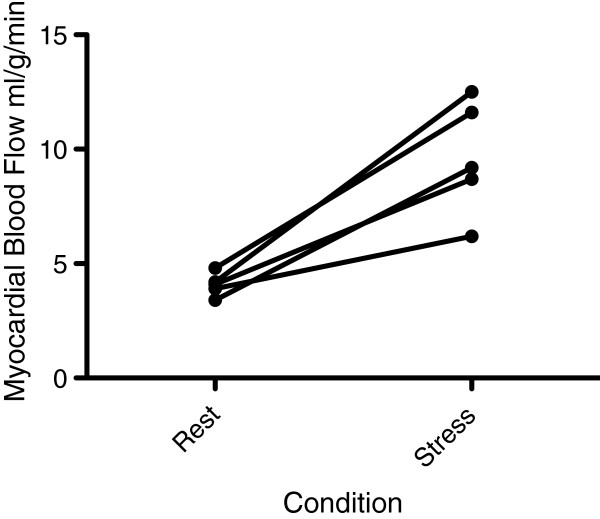
Ladder plots demonstrating the increase in MBF by CMR between rest and dipyridamole stress.

At hyperemic stress, mean LV signal increased from 118 ± 37 before contrast delivery to a peak of 1854 ± 209, resulting in an enhancement ratio of 15.9. Mean myocardial signal increased from 91 ± 34 to 787 ± 187, with an enhancement ratio of 8.0. The mean estimated hyperemic MBF was 9.6 ± 2.5 mL/g/min (Figure 3) (Table 1). There were no significant differences between the four segments. The increase in MBF during stress-induced hyperemia compared with rest MBF was significant (P = 0.0054) with a derived myocardial perfusion reserve of 2.4 ± 0.54.

**Table 1:** MR derived measurements of signal intensity (SI) and perfusion values

**N**	**Scan**	**AIF SI baseline**	**AIF SI max**	**Enhancement ratio**	**LV SI baseline**	**LV SI max**	**Enhancement ratio**	**MBF/ml/g/min**	**MPR**
**1**	stress*	115 ± 24	2220 ± 412	18.30	81 ± 20	945 ± 210	10.67	9.2	2.71
	**rest**	113 ± 27	2199 ± 460	18.46	116 ± 26	650 ± 144	4.60	3.4	
**2**	**stress**	170 ± 36	1780 ± 330	9.47	146 ± 29	1021 ± 243	5.99	8.7	2.12
	rest*	159 ± 29	1602 ± 313	9.08	111 ± 22	440 ± 97	2.96	4.1	
**3**	stress*	83 ± 18	1690 ± 331	19.36	66 ± 15	580 ± 106	7.79	12.5	2.98
	**rest**	138 ± 26	1637 ± 362	10.86	125 ± 26	490 ± 99	2.92	4.2	
**4**	**stress**	137 ± 35	1777 ± 367	11.97	100 ± 23	704 ± 142	6.04	6.2	1.59
	rest*	97 ± 26	1616 ± 369	15.66	122 ± 27	520 ± 102	3.26	3.9	
**5**	stress*	84 ± 19	1804 ± 391	20.48	64 ± 16	684 ± 121	9.69	11.6	2.42
	**rest**	138 ± 22	1832 ± 393	12.28	125 ± 23	489 ± 94	2.91	4.8	
**Mean**	**Stress**	118 ± 37	1854 ± 209	15.92 ± 4.89	91 ± 34	787 ± 187	8.04 ± 2.12	9.64 ± 2.5	2.36 ± 0.54
**Mean**	**Rest**	129 ± 24	1777 ± 254	13.27 ± 3.78	120 ± 6	518 ± 79	3.33 ± 0.72	4.08 ± 0.5	

* denotes which scan was performed first.

*LV* Left ventricle.

*AIF* Arterial input function.

*MBF* Myocardial blood flow.

*MPR* Myocardial perfusion reserve.

### Confocal microscopy

**Figure 4:**
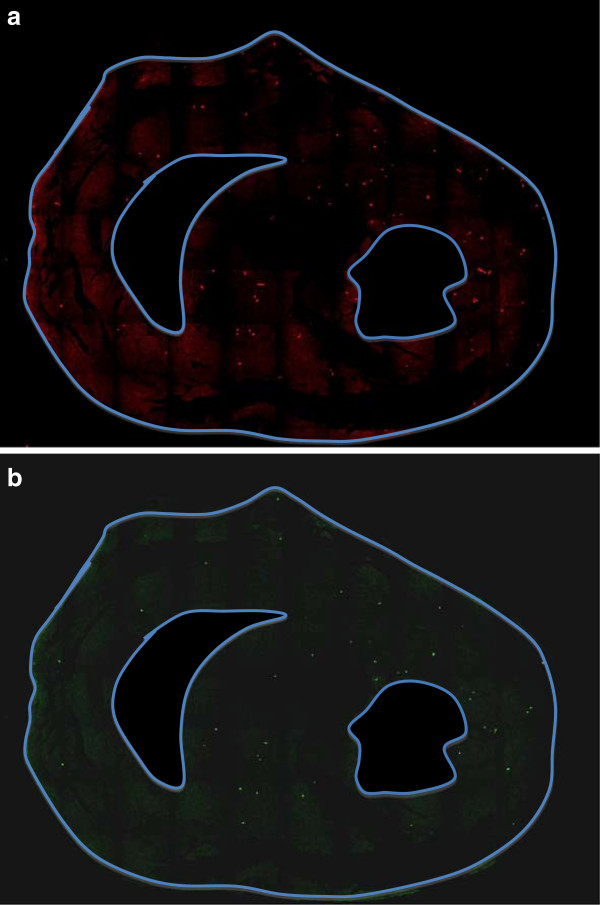
**Confocal microscopy images. a**. confocal images at stress, **b**. confocal images at rest.

**Figure 5:**
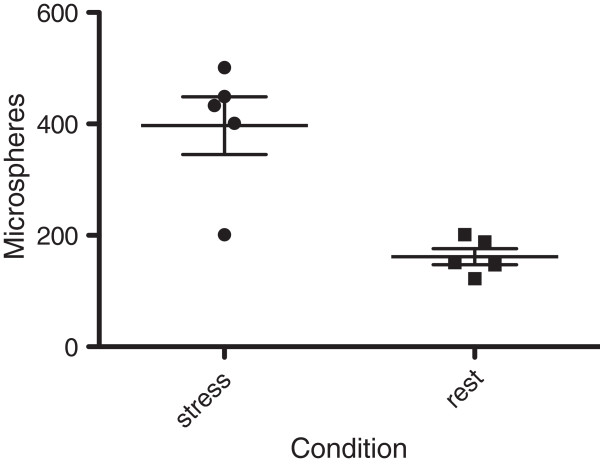
Absolute number of microspheres counted using confocal microscopy at stress and rest using sectioned mid ventricular slices.

**Figure 6:**
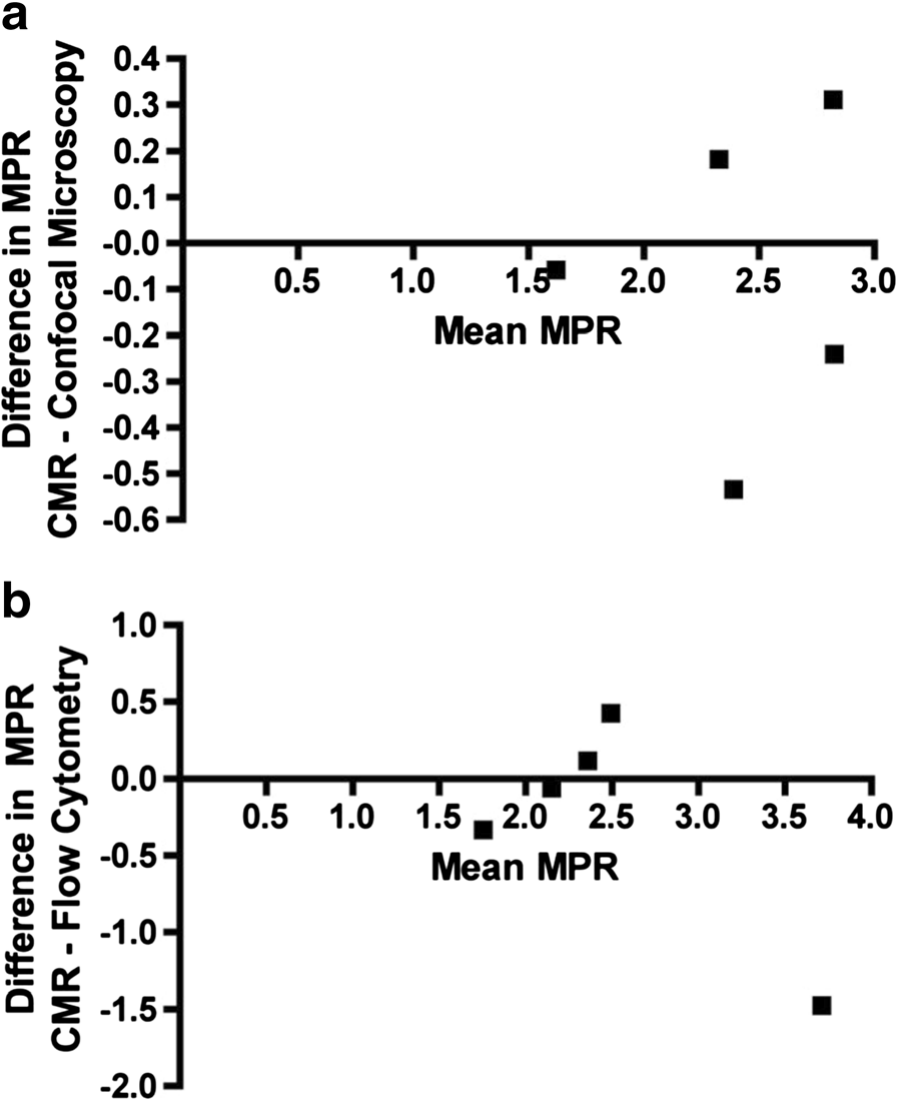
**Bland-Altman images. a**. Bland Altman analysis of MPR (CMR-Confocal Microscopy) **b**. Band Altman analysis of MPR (CMR-Flow Cytometry).

In the midventricular slices matching the perfusion imaging, both fluorescent colored microspheres were identified using confocal laser microscopy (Figures 4a and b). The cumulative mean of microspheres injected at rest was 162 ± 32 and 397 ± 115 at stress (p = 0.0045) (Figure 5), with a mean count ratio of 2.4 ± 0.51. Using Pearson’s correlation, there was a strong association between MPR estimated by CMR and count ratio by confocal microscopy (R = 0.79) and no significant difference was observed. On Bland-Altman analysis, the mean bias between CMR and microscopy was −0.07 (95% limit of agreement −0.73 to 0.59) (Figure 6a).

### Flow cytometry analysis

**Figure 7:**
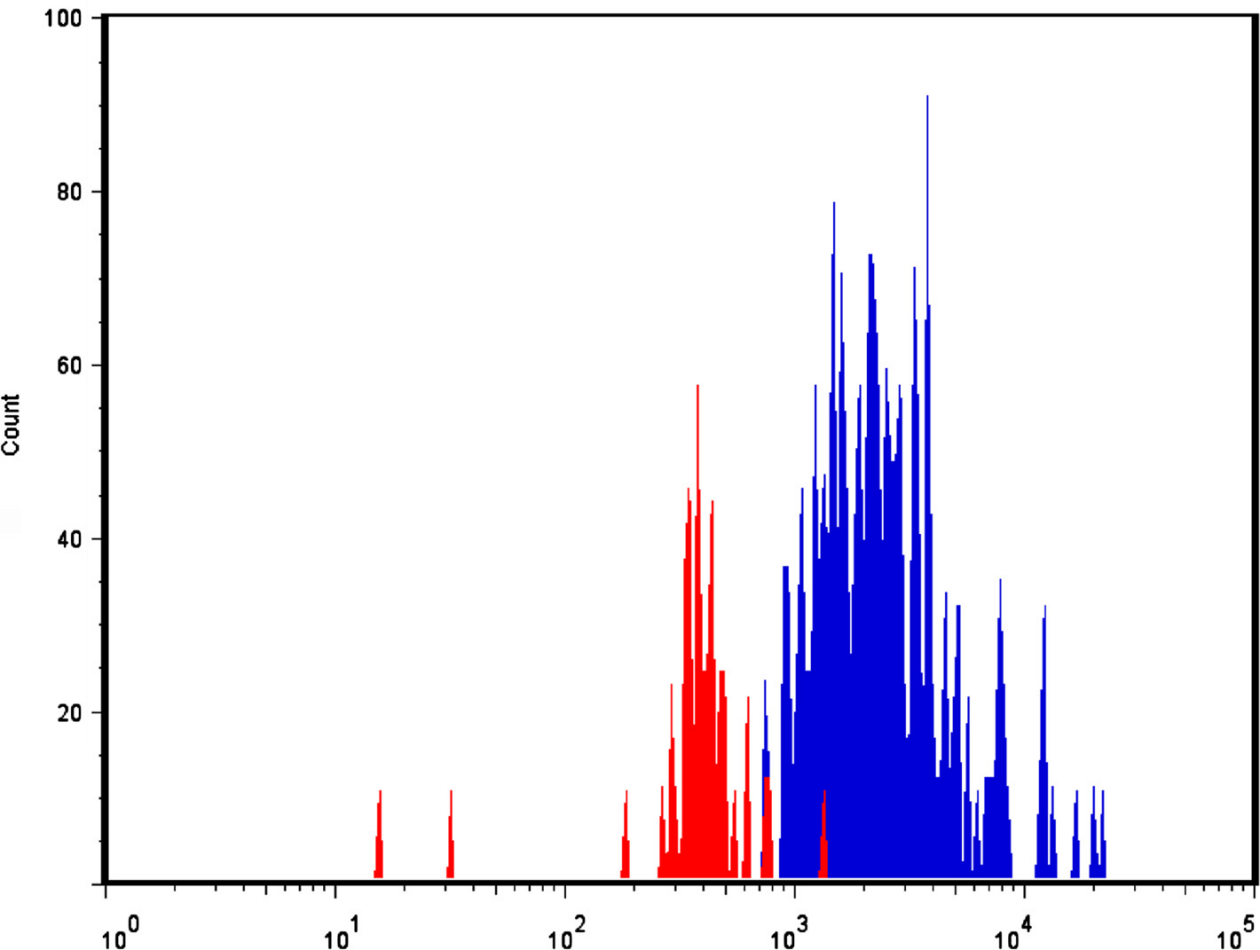
Histogram showing the number of stress (blue) vs rest (red) microspheres in a sample of remaining myocardium showing a relative increased detection of stress to rest microspheres.

**Figure 8:**
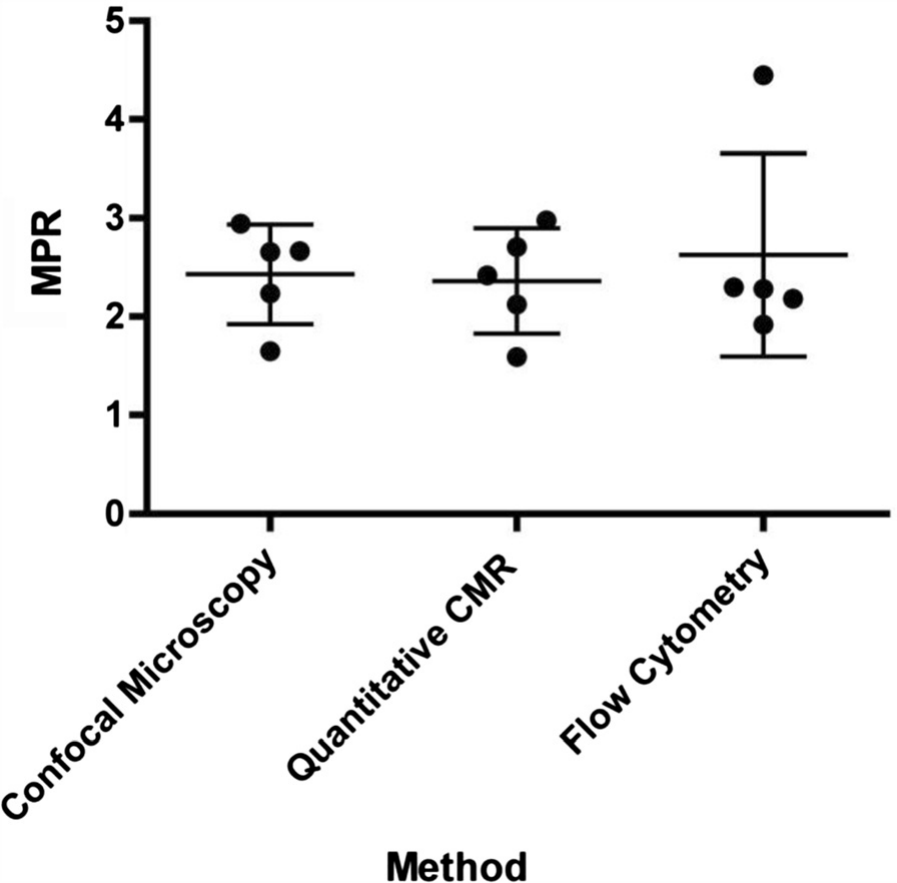
Comparison of the different methods (confocal microscopy, MR and flow cytometry) of assessing perfusion reserve.

The remaining myocardium was digested and sampled for flow cytometry analysis to calculate the relative number of microspheres and to determine a perfusion reserve (Figure 7). The overall mean perfusion reserve was 2.6 ± 1.01, which showed good agreement, but higher variability, compared with the other techniques (Figure 8). Using Pearson’s correlation, there was a strong association between MPR estimated by CMR and count ratio by flow cytometry (R = 0.74) and no significant difference was observed. On Bland-Altman analysis, the mean bias for MPR estimation between CMR and microscopy was −0.26 (95% limit of agreement −1.69 to 0.73) (Figure 6b).

Using ANOVA to compare the perfusion reserve using the different techniques, there was no significant difference between the quantitative CMR data, manual counting of spheres and using confocal microscopy, (P = 0.84, R^2^ = 0.03). Bonferroni post-hoc testing did not show any significant difference between the results derived from different techniques (CMR and histology).

## Discussion

This study has shown that dynamic contrast enhanced myocardial perfusion CMR during dipyridamole-stress in a murine model is feasible at 3 Tesla. MBF and MBF reserve estimates were derived in good agreement with the existing literature and the reference standard of microsphere injection.

The use of first-pass dynamic contrast enhanced myocardial perfusion CMR in rodent models has only recently been reported. The studies published to date used different methods to acquire myocardial perfusion data and in all data were acquired under resting conditions only. Coolen et al [14] performed acquisitions on a 9.4 T Bruker animal scanner. Data acquisition extended over three heart-beats, with a temporal resolution of approximately 400 ms and a 15 ms acquisition window within the cardiac cycle. Acquisition was feasible and detected reduced regional blood flow in an LAD ligation model. This study, however, did not attempt absolute quantification and comparisons with the existing literature could not be made. Makowski et al [15] performed dynamic contrast enhanced myocardial perfusion CMR in five healthy C57BL/6 J mice and four mice with induced myocardial infarction using a similar acquisition method as in our current study. Resting MBF in remote myocardium and control mice was 7.3 ± 0.9 ml/g/min with a reduction in resting MBF in infarcted myocardium matching histology. Nierop et al [13] performed resting imaging in a cohort of nine healthy C57BL/6 mice. They applied a dual-bolus approach for contrast delivery, applied in two separate scans, and demonstrated good repeatability of the method for estimation of resting MBF with a mean MBF of 7.3 ± 0.9 mL/g/min.

Previous studies have derived stress MPR in rodent models using other methods [7,19,20]. Jacquier et al [19] used arterial spin labeling to estimate MBF at rest and during adenosine stress in rats. Because of the long acquisition times of spin labeling techniques, rodents required prolonged anaesthesia and exposure to the stress agent. Moreover, the animals were divided into two separate groups to acquire the rest and stress data. The myocardial perfusion reserve was found to be 2.5 ± 0.6. Raher et al [7] measured rest and adenosine stress myocardial perfusion in wild-type and nitric oxide synthase 3 deficient mice using echocardiography. They found a 2.5 fold increase in MBF estimated with echocardiography and microspheres in the wild-type mice. Murine contrast echocardiography however poses several limitations especially with regards to the spatial resolution, as high frequency probes (14 MHz) preclude the use of harmonic imaging. Furthermore the validation in the study by Raher was not directly performed against closed chest mice and did not quantify absolute blood flow due to the saturation of signal in the LV cavity, which precluded normalization of the blood flow estimate.

The present study used a first pass CMR technique on a clinical scanner with a high spatial (0.2 mm^2^) and temporal resolution (43 msec and acquisition at every heart beat) by using highly accelerated data acquisition. The *k-t* PCA framework for image reconstruction has been shown to improve temporal fidelity and in this study allowed reliable assessment of signal enhancements in the blood pools and in the myocardium and quantitative MBF measurements. The rapid acquisition (<30 seconds) of a first pass method compared with spin labeling methods meant that both stress and rest acquisition could be performed in the same study and in the same animals.

Estimates of both resting and stress in the current study were at the lower end of values reported in the previous literature. We measured a mean resting MBF of 4.1 mL/g/min, compared with previously reported values of 4–7 mL/g/min [8,15,21-24] and a mean stress perfusion MBF of 9.6 ml/g/min compared with a value of 11.5 mL/g/min in the literature [25]. Myocardial perfusion reserve, calculated as the ratio of stress over rest MBF, however was consistent with the literature at 2.4, compared with 2.5 in the studies by both Jacquier [19] and Raher [7] and in excellent agreement with microsphere analysis. There are multiple potential reasons for the relatively low estimates of both resting and stress MBF in the current compared with previous studies, including the age of our murine model, the methods (including anaesthesic regime and greater heat loss from the large bore of clinical scanner over preclinical one, despite having temperature regulation), the acquisition and analysis method used. A small increment in isoflurane from 1.25% to 2% for example has been shown to increase myocardial perfusion [24] by at least two fold. We therefore chose a low isoflurane flow rate. In addition, acquisition with temporal undersampling methods can be associated with low pass filtering effects, which may have led to an underestimation of MBF.

In a recent clinical study [26] in patients with suspected ischaemic heart disease, which used a similar acquisition regime and quantification tool, absolute CMR estimates of MBF were lower than those obtained by PET. Consistent with our preclinical study, both stress and rest perfusion values were lower and cancelled out in the calculation of the MPR.

In both previous studies and our data, MBF reserve was lower than that reported for humans. A potential explanation for this observation is that mice do not increase their oxygen consumption by more than two fold during peak excercise [27]. Oxygen consumption is a major trigger that increases myocardial blood flow [28]. Heart rate (which is predominantly under sympathetic control) does not increase by more than 50% [29] at maximal exercise in mice and therefore may need less coronary reserve than humans to respond to increased myocardial oxygen requirements [30].

In this study, we used dipyridamole as the stress agent. Dipyridamole has previously been used as an alternative or in conjunction with exercise for the evaluation of coronary artery disease in the clinical setting. In our study, dipyridamole caused an insignificant increase in heart rate and the slow injection regime was well tolerated. Compared with adenosine, dipyridamole has the advantage that it can be administered prior to contrast agent administration, so that only one venous access is required. However, our data show a variable response to dypyridamole in the five animals and it is noted that it may be less potent than adenosine [31]. More recently, the stress agent regadenoson has become available for clinical use. This agent was originally validated in rats and is reported to have fewer side effects than adenosine and to cause more marked hyperemia [32]. Like dipyridamole it can be infused as a bolus prior to giving the contrast agent. Future studies are warranted to test this agent, although questions still remain as the optimal timing with injection in as the hyperemic response is not as sustained as with dipyridamole.

In this study absolute perfusion was calculated by Fermi-deconvolution of the myocardial signal response with the arterial input function and applying previously described methods [17]. An important assumption for this technique is the linearity between signal intensity and contrast agent concentration. Particularly for the arterial input function this condition is not easily met because of saturation of the signal by high contrast agent concentrations in the blood. To overcome this problem for the second injection of contrast we applied baseline correction [33] and randomized the order of rest and stress acquisitions, but saturation effects especially in the input function may have underestimated MBF. Other methods using a dual bolus strategy have been reported mainly in clinical studies. One recent report by van Nierop acquired dual bolus data under resting conditions and in two separate settings [13]. The method is challenging to perform in rodent models in addition to stress, but may present a future option for improved MBF estimation. Both single-bolus and dual-bolus perfusion methods have been shown to correlate closely with MBF in particular when myocardial perfusion is normal [34].

### Limitations

Contrast and stress agent were delivered by hand. This process can in principle be automated to improve reproducibility. Our perfusion method acquires only a single slice, but more slices could be acquired at the expense of temporal resolution or in consecutive experiments. We did not perform true quantification of absolute MBF by microspheres because there are concerns about the validity of this technique in rodents [35]. We were therefore not able to establish if CMR systematically underestimated MBF, but were able to demonstrate good agreement for the more robust MBF reserve. Further refinements of the quantitative tool may be required for estimation of absolute MBF in murine models, however values for MPR appear to be consistent with the literature. Few investigators have used the microsphere method using the reference sample approach for absolute quantification [21,22,36,37].

## Conclusions

Dynamic contrast enhanced myocardial perfusion CMR during hyperemic stress in a mouse model is feasible. In this study, data were acquired on a 3 Tesla scanner using an approach similar to clinical acquisition protocols, thus facilitating translation of imaging findings between rodent and human studies. This may in future help elucidate mechanisms and develop therapies for cardiovascular disease. The rapid data acquisition of dynamic contrast enhanced myocardial perfusion CMR compared with arterial spin labeling will facilitate its incorporation into more comprehensive in vivo CMR protocols.

## Abbreviations

CMR: Cardiovascular magnetic resonance; CAD: Coronary artery disease; MBF: Myocardial blood flow; MPR: Myocardial perfusion reserve

## Competing interests

SP is funded by British Heart Foundation fellowship FS/10/62/28409 and receives research grant support from Philips Healthcare.

SK receives funding from the Swiss National Science Foundation (grant number CR3213_132671/1) and research support from Bayer (Switzerland).

EN receives grant support from Bayer Healthcare and Philips Healthcare.

## Authors’ contributions

RJ designed the study protocol, carried out the MR studies, analyzed the data and drafted the manuscript. SK designed and updated the perfusion sequence and helped to draft the manuscript. MM, AP, ASP, CJ and RB participated in the study design, helped to perform the MR studies and perform analysis of the results. AC and NZ performed the MRI quantification analysis of the results, together with Professor EN helped draft the manuscript. Professor SP is the chief investigator and conceived the idea of this murine stress perfusion protocol, analyzed the data and drafted the manuscript. All authors read and approved the final manuscript.
